# Methods, Diagnostic Criteria, Cutoff Points, and Prevalence of Sarcopenia among Older People

**DOI:** 10.1155/2014/231312

**Published:** 2014-12-17

**Authors:** Valéria Pagotto, Erika Aparecida Silveira

**Affiliations:** ^1^Nursing School, Federal University of Goiás, 227 Street, Block 68, s/n, Setor Leste Universitário, 74605-080 Goiânia, GO, Brazil; ^2^School of Medicine, Federal University of Goiás, Postgraduate Program in Health Sciences, 235 Street s/n, Setor Leste Universitário, 74605-020 Goiânia, GO, Brazil

## Abstract

*Aim*. To identify methods, index, diagnostic criteria, and corresponding cutoff points used to estimate the prevalence of sarcopenia in older people in different countries. *Methods*. A systematic review was carried out in accordance with PRISMA Statement. The search encompassed the MEDLINE and LILACS databases and was executed during March 2012 using the keyword sarcopenia. *Results*. A total of 671 studies were identified by the search strategy, and 30 meet all inclusion criteria. Specifically for dual-X-ray absorptiometry, prevalence ranged from 2.2% to 95% in men and from 0.1% to 33.9% in women. For bioelectrical impedance analysis, the range was from 6.2% to 85.4% in men and 2.8% to 23.6% in women. Regarding anthropometric and computed tomography, prevalence rates were, respectively, 14.1% and 55.9%. *Conclusions*. Heterogeneity in prevalence of sarcopenia was identified, due to diagnostic method choice, cutoff points, and, characteristics of the population as well as reference population. These factors should be considered in research designs to enable comparison and validation of results. Despite the limitations of most studies that indicated high prevalence rates, the results indicate the need for early detection of this syndrome.

## 1. Introduction

Sarcopenia was conceptualized in the last two decades [1] and since then, several studies have been carried out as attempts to clarify definitions for estimation of the issue in the elderly, resulting in a wide diversity of methods and diagnostic criteria [2–4]. As a consequence of such diversity, international research groups have proposed definitions to enable the recommendation of parameters used in the evaluation of sarcopenia [5–8]. The Working Group on Sarcopenia in Older People (EWGSOP) defined it as a syndrome characterized by the progressive and generalized loss of muscle mass, strength, and performance [6]. In 2011, the International Working Group on Sarcopenia (IWGS) defined it as the loss of skeletal muscle mass and strength, associated with the aging process [7].

The study of sarcopenia is important in the areas of public health, geriatrics, and gerontology due to its contribution to adverse outcomes in the elderly [4, 9, 10], hospitalizations [10], and early death [11]. However, knowledge on the magnitude of sarcopenia in the elderly population is limited or at least controversial due to the variety of definitions and diagnostic parameters utilized [3–8, 12]. We did not find systematic reviews focusing on the analysis of sarcopenia magnitude throughout different continents, nor on the different definitions and diagnostic methods for muscle mass evaluation. Analysis of existing studies, including a comparison of the aforementioned aspects, can contribute to the knowledge base on the use of methods and diagnostic criteria and even help direct towards a more operational and less theoretical definition of sarcopenia not only in clinical-epidemiological research but also for health services. Such research can contribute to the efforts to standardize diagnostic criteria applied in different continents and establish the magnitude of sarcopenia in the elderly.

Therefore, the objective of this study was to identify the methods, index, diagnostic criteria, and corresponding cutoff points used to estimate the prevalence of sarcopenia in older people in different countries, defining global panorama of the issue.

## 2. Methods

A systematic review was carried out following the recommendations for reporting systematic reviews and meta-analyses of the Preferred Reporting Items for Systematic Reviews and Meta-Analyses (The PRISMA Statement) [13].

Searches were carried out in the MEDLINE and LILACS databases until December, 2012, with no restriction on year of publication. Sarcopenia was the only keyword used due to the variety of methods for diagnosing muscle mass, strength, and performance. The following search limits were established: research on humans, in English, French, German, Spanish, or Portuguese, and age group over 45 years, with the keyword in any field. Within the search results obtained, manual searches were then carried out on the bibliographic references cited within the articles.

Subsequently for the identification of studies in the databases, duplicates were excluded and the titles and abstracts of the remaining results were screened, following the eligibility criteria: inclusion of prevalence rates of sarcopenia as well as the methods for measuring muscle mass and for diagnosing sarcopenia. All titles and abstracts were independently screened by two authors. The eligible articles were read in full and those that met all criteria were included.

A tool was developed to analyze data, which contained the following information: authors, year of publication, geographical location of the population, study type, sample size, age range, measurements and techniques for diagnosis of sarcopenia, population used as reference to compare muscle mass, type of evaluation method for muscle mass, indices, diagnostic criteria, cutoff points, cutoff values, and prevalence according to each technique by sex and number of men and women in each study. The confidence intervals (95% CI) were calculated in STATA 12.0 and were presented in graph-type high-low. If the article did not have the information for calculating the CI, we contacted the corresponding author by e-mail requesting the data. Due to heterogeneity between studies, meta-analysis was not performed.

To assess the quality of articles, we adopted the methodology proposed by Downs and Black [14], whose purpose is to guide auditors in identifying the methodological features of most relevant observational studies. The proposed score is composed of 27 questions that assess clarity of writing (nine items), external validity (two items), internal validity (seven items), confounders (four items), and power of the study (one item). This tool was adapted as described by Monteiro and Victora [15]; because those criteria were originally designed for the evaluation of clinical trials being excluded four questions apply only to this type of study. Thus, the maximum possible score for each item was 24.

## 3. Results

**Figure 1:**
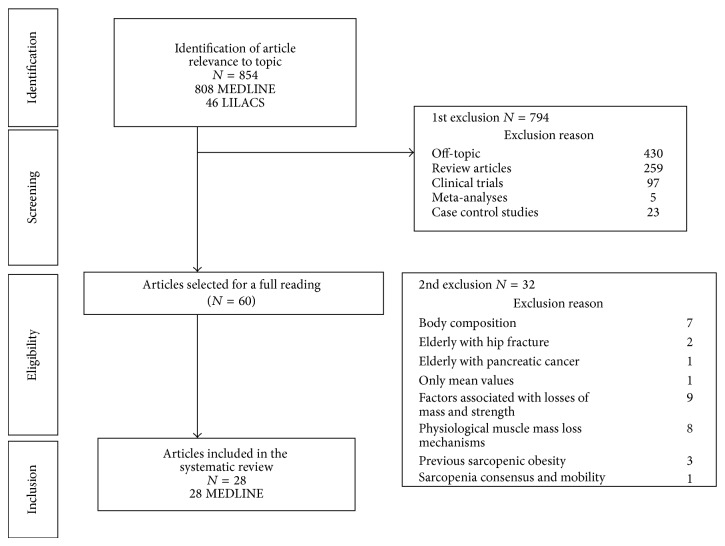
Flow diagram for identification, screening, eligibility, and inclusion of articles in systematic review.

Through the utilization of search strategies, 854 unduplicated articles were identified: 808 in MEDLINE and 46 in LILACS. After reading of titles and abstracts, 794 articles were excluded, of which 430 were off-topic and 384 were excluded due to the type of study (review, meta-analysis, clinical trials, and case control). Sixty articles were eligible for a full reading, and after a second round of exclusions, the final sample size was of 28 articles (Figure 1). Critical appraisal of the studies included in our analysis revealed that they were of high quality and credibility.

The mean score of methodological quality was 17.5. Regarding the general characteristics of the articles, cross-sectional studies were predominant (60%), with publications dating from 1998 to 2012. Ten studies were conducted in Asia, nine in North America, and eight in Europe and one was conducted in South America (Brazil) (Table 1).

Twenty-six studies (92.8%) used exclusively muscle mass for the definition of sarcopenia, while two studies (7.2%) included mass, strength, and performance, as recommended by the European Sarcopenia Consensus. For this reason we decided to evaluate only muscle mass for comparison purposes (Tables 1, 2, and 3).

To estimate muscle mass, eighteen studies (64.3%) used dual-energy X-ray absorptiometry (DXA) (Table 1), eight used bioelectrical impedance analysis (BIA) (Table 2), and two used anthropometric measurements (calf circumference) (Table 3).

Within the DXA studies, all 18 used the appendicular muscle mass index (AMMI), defined as the sum of fat-free arm and leg mass in kg (appendicular muscle mass, AMM) divided by the square of the height in meters (AMMI = AMM/height^2^). Nine of the studies compared AMMI with other indices: three with total muscle mass (TMM), defined as AMM × 1.33/height^2^, and five with mass from a regression analysis adjusting fat mass and height (Table 1).

Three different BIA indices were found. The skeletal muscle index (SMI) adjusted for squared height was used in six studies and adjusted for weight multiplied by 100 was used in three studies (37.5%). For the calculation of SMI, muscle mass was estimated by the equation: Skeletal Muscle Mass (SMI) = [(Height^2^/Resistance × 0.401) + (sex × 3.825) + (age ×  −0.071)] + 5, where height is given in cm and resistance in ohms, female = 0, male = 1, and age is expressed in years (Bahat et al. 2010). Only one index estimated muscle mass using the DuBois formula: Body Surface Area (BSA) = (kg^0.425^ × m^0.725^) × 0.007184 (Table 2).

The two anthropometric studies measured muscle mass using calf circumference, with a cutoff point of 31 cm (Table 3).

Four different criteria for sarcopenia diagnosis were identified: sarcopenia was defined when AMM was two standard deviations (SD) below the mean of a young reference population, by sex (20 studies), or when 20th percentile was below the elderly sample distribution (3 studies); sarcopenia was defined by the residual method (5 studies); and finally using a cutoff point by Roc curve analysis. Seven studies used the reference population of the USA. Rosetta study has a cutoff point of 7.26 kg/m^2^ for men and 5.45 kg/m^2^ for women. The other studies used their own young population, with ages ranging from 18 to 40 years. Three studies classified sarcopenia into Class 1 for muscle mass between −1 and −2 standard deviations from the mean and Class 2 for muscle mass below −2 standard deviations from the mean, both for their reference population. The variations in cutoff points for estimation of muscle mass are shown in Tables 1, 2, and 3.

**Figure 2:**
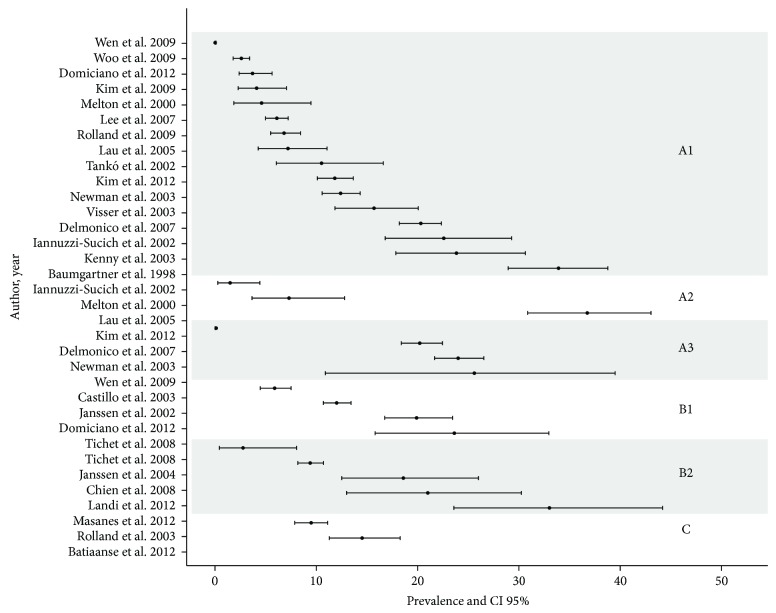
Sarcopenia prevalence and CI 95% in women. (A: DEXA; A1: appendicular skeletal muscle mass index (AMMI); A2: total skeletal muscle mass index (AMMT); and A3: residuals method; B: BIA; B1: skeletal muscle index/height; B2: skeletal muscle index/weight; C: calf circumference).

**Figure 3:**
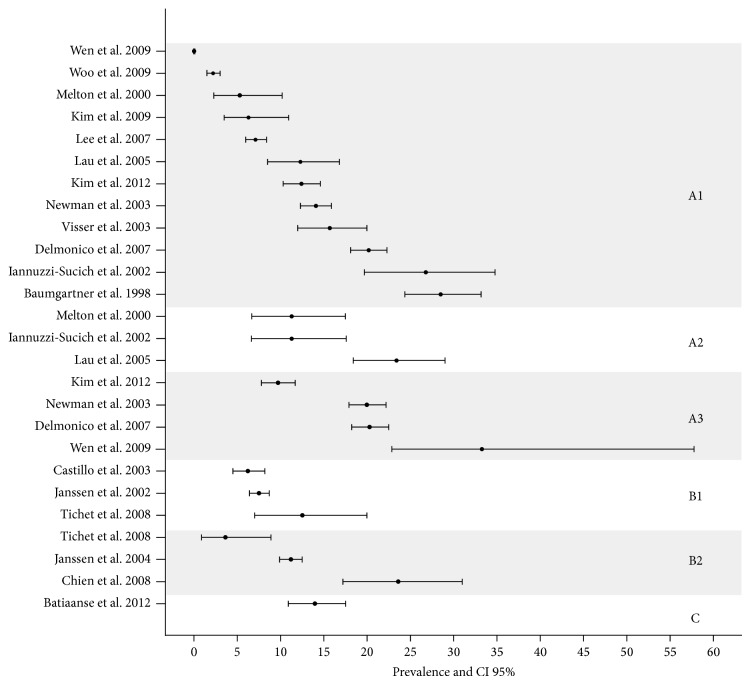
Sarcopenia prevalence and CI 95% in men. (A: DEXA; A1: appendicular skeletal muscle mass index (AMMI); A2: total skeletal muscle mass index (AMMT); A3: residuals method; B: BIA; B1: skeletal muscle index/height; B2: skeletal muscle index/weight; C: calf circumference).

Considering all methods and diagnostic criteria, prevalence of sarcopenia in the elderly ranged from 0.0% to 85.4% in men and 0.1% to 33.6% in women. For DXA, prevalence ranged from 0.0% to 56.7% in men and 0.1% to 33.9% in women (Table 1). For BIA, the range was from 6.2% to 85.4% and from 2.8% to 23.6%, in men and women, respectively (Table 2). Figures 2 and 3 summarize all prevalence and confidence intervals (95%) of 21 studies for men and 25 studies for women.

## 4. Discussion

This systematic review provides a broad panorama of sarcopenia prevalence in elderly people from five continents, allowing for comparisons of different diagnostic methods and cutoff points, thus contributes to defining the magnitude of the problem in different parts of the world, highlighting lacunae in some geographic areas and the lack of uniformity in diagnostic criteria, and so encourages reflections and propositions on the study of sarcopenia.

The first sarcopenia prevalence studies were only published 10 years after the term was coined in 1989. The first index proposed for diagnosing sarcopenia by muscle mass was the appendicular muscle mass index (AMMI), which is currently widely used in studies from different countries [12, 22, 23, 25, 26, 31, 41, 42]. When using AMMI, muscle mass is measured by DXA (kg) and the result is compared to a young reference population [4].

The use of the AMMI classification criterion for other populations (*n* = 18) has provided a wide range of prevalence, varying from 0.0% to 56.7% in men and from 0.0% to 33.9% in women. These results can be attributed to racial characteristics, highlighting physical constitution and cultural aspects that imply physical activity levels, dietary regimes, and life quality of the elderly in different countries. This can be exemplified by the low prevalence encountered among the Chinese population, [25, 26, 41] leading to the authors conclusion that AMMI is not an appropriate method to diagnose sarcopenia in this specific population. The cutoff points for the Chinese [41] population are lower than for Americans [4] (<5.72 versus 7.26 in men and <4.82 versus <5.45 in women, resp., for Chinese and North-Americans), with young people of the same ethnic group as reference. The mean AMMI of young Asians was approximately 15% lower than that of Caucasians even after height adjustments [22, 41]. Therefore, low muscle mass in young Asians will result in lower prevalence rates in the elderly. Moreover, sarcopenia may be less prevalent in Asians due to differences in risk factors such as a better dietary profile and higher levels of physical activity than Western populations, which act as protective factors against sarcopenia [26].

Although several studies apply AMMI (*n* = 15) and the recommendations to use it [6], other criteria and indices have been proposed to diagnose sarcopenia. Newman et al. [12] proposed a criterion based on the appendicular muscle mass adjusted for body weight (fat mass) and height, where the cutoff point was the 20th percentile of the distribution of linear regression residuals. This method was used in five studies [12, 23, 26, 41, 42] and presented better sensitivity in the identification of sarcopenic individuals, particularly among elderly patients with a high prevalence of overweight and obesity [31, 37]. This method is recommended for sarcopenia studies, in overweight and/or obese populations [12].

Melton III et al. [16] developed the TMMI, which was used in four studies [16, 18, 22, 41]. This index also shows important differences in prevalence due to the same factors that explain the variation in prevalence with AMMI. As TMMI identifies the total muscle content, the prevalence of sarcopenia could be higher in comparison with AMMI since appendicular muscle mass represents 75% of total body muscle mass [16, 43]. However, prevalence measured by TMMI was lower than that calculated by AMMI and is justified by errors in the estimation of total muscle mass, such as a potential overstatement of water or fat contents, which limit the usefulness of TMMI [43].

Although DXA is precise and is recommended, muscle mass was validated through other measurements in order to enable operationalization and applicability to clinical settings, such as electrical impedance (BIA) and anthropometric measurements. Starting from BIA, Janssen et al. [32] proposed that SMI be adjusted both for height and for weight. Sarcopenia prevalence according to this method also presented significant differences, [33–35] attributed to the different characteristics of study populations and cutoff point references, as well as to the inherent limitations of BIA, which presents a standard error of 9% [32] in the estimation of muscle mass. The increase in total body water, particularly extracellular water, may result in underestimation of fat body mass and overestimation of lean body mass [3].

Less frequently (*n* = 3) the anthropometric measurement was also utilized to evaluate muscle mass and diagnose sarcopenia, due to the low cost, noninvasive character, and basic training requirements. Prevalence found through this method was significantly lower than that obtained with DXA or BIA [39, 40].

From the 28 articles selected for review, four types of cutoff points for sarcopenia diagnosis were identified, being two standard deviations below the mean for a young reference population, the most used cutoff point (*n* = 17), despite its limitations. Only one cohort study defined sarcopenia as a loss of 3% of baseline AMM, based on the coefficient of variation for the measurement of AMM using DXA, which was 2-3% [21]. Visser [44] points out that most definitions include a cutoff point for low muscle mass, but not for loss of muscle mass. The statement of sarcopenia refers to a relative deficiency in muscle mass and does not specify loss [44]. At this point it is discussed that the comparison with elderly population of the same population, noninstitutionalized and with high life quality standards, could reflect with greater precision the deficiency of muscle mass instead of the comparison with young population. The affirmation is supported by studies that show that after the age of 30, the musculoskeletal system starts to undergo a progressive loss, with a 1-2% decrease in muscle mass starting at the age of 50, which becomes more pronounced after the age of 60 [45]. Caution must be exercised when making comparisons with a young population, as young people have not been exposed to the same factors that older people have experienced throughout their lives, besides the progressive loss of muscle mass that is characteristic of the natural aging process. Thus, studies [46] on the causes of sarcopenia evaluate a wide variety of conditions that go beyond known risk factors, such as sedentary lifestyles, dietary intake, influence of hormones, and cytokine levels, supporting the definition of sarcopenia as a geriatric syndrome.

Despite the differences encountered between the studies, regarding methods and definitions for estimating muscle mass, the present study demonstrates that a substantial proportion of the elderly population has sarcopenia, even in healthy populations. It is questioned, however, what the acceptable progression of loss of muscle mass is as a consequence of the aging progress and what values can identify a harmful loss; that is, that could place the elderly at risk of falling, dependence, and frailty syndrome. These are questions that can direct future research and therefore prospective studies are required and recommended to delineate the natural progression of sarcopenia and its predispositional factors.

The evaluation of sarcopenia, as proposed by the first definitions and by the EWGSOP and IWGS consensus, has been performed in research, even with its inherent limitations. The use of DXA for the estimation of muscle mass guarantees higher reliability and must be the method chosen to evaluate muscle mass in research and for patients of higher clinical complexity. DXA, however, is of difficult operationalization and access in the health service routine, due to elevated cost and specialized professional requirements. It is recommended that other methods, such as BIA and CC, be developed and validated by research devoted to the tracking and consequently to the screening of sarcopenia in health services, due to easiness of application and low cost.

In conclusion, more than one operational definition, it is necessary that the current methods are applied in clinical practice, because sarcopenia presents low visibility in the health services and has not achieved the same space in clinical settings as in research. Therefore, propagation among geriatrics and gerontology healthcare professionals is important and must be included in the context of public health politics.

## Figures and Tables

**Table 1:** Diagnostic criteria and prevalence of sarcopenia according to gender estimated by Dual-X-ray-absorptiometry (DXA) method (*n* = 18).

	Authors, y	Country	Study design (*n*)	Age (y)	Diagnostic criteria				Prevalence (%)	
					Index^∗^	Description and reference population	Cutoff points			
							Male (kg/m^2^)	Female (kg/m^2^)	Male (%)	Female (%)
1	Baumgartner et al. 1998 [4]	USA	Cross-sectional Population-based study (808)	>60	AMMI	−2 SD below gender-specific mean of young adults from Rosseta study (18–40 y)	7.26	5.45	28.5	33.9

2	Melton III et al. 2000 [16]	USA	Cross-sectional Population-based study (300) (community-dwelling)	≥60	TSMI	−2 SD below gender-specific mean of young adults from Rochester (20–50 y)	6.77	4.51	11.3	7.3
					AMMI	−2 SD below gender-specific mean of young adults from Rosseta study (18–40 y)	7.26	5.45	5.3	4.6

3	Tankó et al. 2002 [17]	Denmark	Cross-sectional (754 healthy women)	>60	AMMI	−2 SD below the normal sex-specific means for young persons of Danish population (19–39 y)	—	5.40	—	12.3

4	Iannuzzi-Sucich et al. 2002 [18]	USA	Cross-sectional Community-dwelling (337)	≥65	AMMI	−2 SD below gender-specific mean of young adults from Rosseta study (18–40 y)	7.26	5.45	26.8	22.6
					TSMI	−2 SD below gender-specific mean of young adults from Rochester (18–40 y)	6.77	4.51	11.3	1.5

5	Kenny et al. 2003 [19]	USA	Cross-sectional Women users ERT (189)	>59	AMMI	−2 SD below gender-specific mean of young adults from Rosseta study (18–40 y)	—	5.45	—	23.8

6	Gillette-Guyonnet et al. 2003 [20]	France	Cohort EPIDOS (1.321 women)	75–80	AMMI	−2 SD below gender-specific mean of young adults from Rosseta study (18–40 y)	—	5.45	—	8.9

7	Visser et al. 2003 [21]	Amsterdam	Cohort LASA (1.008)	55–85	MMA	Lowest sex-specific 15th percentile of the cohort (loss muscle mass greater than 3%)	—	—	15.7	

8	Newman et al. 2003 [12]	USA	Cohort (2.984) Health ABC Study	70–79	AMMI	<20th percentile sex-specific distribution	7.23	5.67	14.1	12.4
					AMMI^R^	<20th percentile sex-specific residuals distribution adjusted for fat mass and height	−2.29^†^	−1.73^†^	20.0	24.0

9	Lau et al. 2005 [22]	China	Cross-sectional (527) Community-dwelling	>70	AMMI	−2 SD below gender-specific mean of Chinese young adults (20–40 y)	<5.72	<4.82	12.3	7.2
					TSMI	−2 SD below gender-specific mean of Chinese young adults (20–40 y)	9.9	8.5	23.4	36.7

10	Delmonico et al. 2007 [23]	USA	Cohort (2.976) Health ABC Study	70–79	AMMI	<20th percentile sex-specific distribution	7.25	5.67	20.3	20.2
					AMMI^R^	<20th percentile sex-specific residuals distribution adjusted for fat mass and height	—	—	20.2	20.3

11	Lee et al. 2008 [24]	China	Cross-sectional (4.000) Community-dwelling	≥65	AMMI	−2 SD below gender-specific mean of Chinese young adults (20–40 y)	7.19	6.05	7.1	6.1

12	Kim et al. 2010 [25]	Korea	KSOS (526) Community-dwelling	≥60	AMMI	−2 SD below gender-specific mean of Korean young adults (20–39 y)	6.58	4.59	6.3	4.1

13	Woo et al. 2009 [26]	China	Cohort (3.153) Community-dwelling	≥65	AMMI	−2 SD below gender-specific mean of Chinese young adults (20–40 y)	<7.4	<6.4	2.2	2.6

14	Rolland et al. 2009 [27]	France	Cohort/ EPIDOS (1.308) Community-dwelling women	≥75	AMMI	−2 SD below gender-specific mean of young adults from Rosseta study (18–40 y)	—	5.45	—	6.8

15	Sanada et al. 2010 [28]	Japan	Cross-sectional 1488 Community-dwelling	70–85	AMMI	Class 1: −1 to 2 SD below gender-specific mean of Japanese young adults (18–40 y)	7.77	6.12	6.7	6.3
						Class 2: −2 SD below gender-specific mean of Japanese young adults (18–40 y)	6.87	5.46	56.7	33.6

16	Wen et al. 2011 [29]	China	Cross-sectional (72) Community-dwelling	60–69	AMMI	−2 SD below gender-specific mean of Chinese young adults (20–40 y)	<5.85	<4.23	0.0	0.0
					AMMI^R^	<20th percentile sex-specific residuals distribution adjusted for fat mass and height			33.3	25.6

17	Kim et al. 2012^∗^ [30]	Korea	Cross-sectional KNHANES IV (2008-2009) 2.332	≥65	AMMI	Class 1: −1 to 2 SD below gender-specific mean of Korean young adults (20–39 y)	7.50	5.38	30.8	10.2
						Class 2*: *−2 SD below gender-specific mean of Korean young adults (20–39 y)	6.58	4.59	12.4	0.1
					AMMI^R^	Class 1: −1 to 2 SD below gender-specific mean of Korean young adults (20–39 y)	32.2%	25.6%	29.5	30.3
						Class 2: −2 SD below gender-specific mean of Korean young adults (20–39 y)	29.1%	23.0%	9.7	11.8

18	Domiciano et al. 2013 [31]	Brazil	Cross-sectional (611 women community-dwelling)	≥65	AMMI	−2 SD below gender-specific mean of young adults from Rosseta study (18–40 y)	—	5.5	—	3.7
					AMMI^R^	<20th percentile sex-specific residuals distribution adjusted for fat mass and height	—	−1.45^†^	—	19.9

^*^AMMI: appendicular skeletal muscle mass index (appendicular skeletal muscle mass/height^2^).

^*^TSMI: total skeletal muscle mass index (total skeletal muscle mass/height^2^).

^*^AMMI^R^ = appendicular skeletal muscle mass index: regression among appendicular skeletal muscle mass. Height and body fat (residuals method).

^†^Residuals method.

EPIDOS: Epidemiologie de l'Osteoporose Study.

LASA: longitudinal aging study Amsterdam.

Health ABC Study: The Health, Aging, and Body Composition Study.

KSOS: Korean Sarcopenic Obesity Study.

KNHANES IV: Fourth Korean National Health and Nutritional Examination Surveys.

**Table 2:** Diagnostic criteria and prevalence of sarcopenia according to gender estimated by bioelectrical impedance analysis (BIA) method (*n* = 8).

	Authors, y	Country	Study design (*N*)	Age (y)	Diagnostic criteria				Prevalence (%)	
					Index^∗^	Description and reference population	Cutoff points			
							Male (kg/m^2^)	Female (kg/m^2^)	Male (%)	Female (%)
1	Janssen et al. 2002 [32]	USA	Cross-sectional NHANES III (4.504)	≥60	SMI^%^	Class 1: −1 to 2 SD below gender-specific mean of young adults (18–39 y)	37–31%	28–22%	44.0	60.3
						Class 2: −2 SD below gender-specific mean of young adults (18–39 y)	<31%	<22%	7.5	12.2

2	Castillo et al. 2003 [33]	USA	Cross-sectional (1.700) community-dwelling	55–98	SMI^%^	−2 SD below gender-specific mean of young adults from Pichard study	47.9%	34.7%	6.2	5.9

3	Janssen et al. 2004 [9]	USA	Cohort NHANES III (4.499)	≥60	SMI^kg/m2^	Based on ROC curve analysis for moderate dysfunction	8.51–10.75	5.76–6.75	53.1	21.9
						Based on ROC curve analysis for elevated dysfunction	≤8.50	≤5.75	11.2	9.4

4	Chien et al. 2008 [34]	Taiwan	Cross-sectional (302) community-dwelling	≥65	SMI^kg/m2^	−2 SD below gender-specific mean of Taiwanese young adults (18 a 40 y)	<8.87	<6.42	23.6	18.6

5	Tichet et al. 2008 [35]	France	Cross-sectional (218) volunteers of healthcare centers	60–78	SMI^%^	−2 SD below gender-specific mean of French young adults (18 a 39 y)	34.4%	26.6%	12.5	23.6
					SMI^kg/m2^	−2 SD below gender-specific mean of young adults (18 a 39 y)	8.6	6.2	3.6	2.8

6	Bahat et al. 2010 [36]	Turkey	Cohort (217) male nursing home residents	>60	ASC	−2 SD below gender-specific mean of young adults from control group (24–45 y).	29.6 kg/ASC	—	85.4	—

7	Landi et al. 2012 [10, 37]	Italy	Cross-sectional (122) Nursing home residents	>70	SMI (kg/m^2^)	−2 SD below gender-specific mean of Italian young adults (18 a 40 y)	<8.87	<6.42	68.0	21.0

8	Masanes et al. 2012 [38]	Spain	Cohort (200)	70–80		−2 SD below gender-specific mean of Spanish young adults (20 a 40 y)	<8.25	6.68	10.0	33.0

^*^SMI^kg/m2^: skeletal muscle index: muscle mass calculated using the bioelectrical impedance analysis equation of Janssen et al. (2000) [3]/height^2^.

^*^SMI^%^: skeletal muscle index: muscle mass calculated using the bioelectrical impedance analysis equation of Janssen et al. (2000) [3]/weight × 100.

NHANES III: National Health and Nutritional Examination Surveys.

**Table 3:** Diagnostic criteria and prevalence of sarcopenia according to gender estimated by anthropometry (*n* = 2).

	Authors, y	Country	Study design (*N*)	Age (y)	Diagnostic criteria				Prevalence (%)	
					Index	Description and reference population	Cutoff points			
							Male (kg/m^2^)	Female (kg/m^2^)	Male (%)	Female (%)
**Anthropometry**										

1	Rolland et al. 2003 [39]	France	Cross-sectional (1311) community-dwelling women	≥70	CC	31 cm	—	31 cm	—	9.5

2	Bastiaanse et al. 2012^∗^ [40]	The Netherlands	Transversal 884 intellectual disabilities	≥50	CC	31 cm	31 cm	31 cm	14.0	14.5

CC: calf circumference.

^*^Bastiaanse et al. 2012 [40]: EWGSOP criteria. The prevalence using only muscle mass estimated by CC was 9.1% in both sexes.

EPIDOS: European Patient Information and Documentation Systems (EPIDOS) Study.
